# Evaluation of Task fMRI Decoding With Deep Learning on a Small Sample Dataset

**DOI:** 10.3389/fninf.2021.577451

**Published:** 2021-02-12

**Authors:** Sunao Yotsutsuji, Miaomei Lei, Hiroyuki Akama

**Affiliations:** ^1^School of Life Science and Technology, Tokyo Institute of Technology, Tokyo, Japan; ^2^Ex-Graduate School of Science and Technology, Tokyo Institute of Technology, Tokyo, Japan; ^3^Institute of Liberal Arts, Tokyo Institute of Technology, Tokyo, Japan

**Keywords:** brain decoding, cross-subject modeling, cross-validation, deep learning, fMRI, model selection, MVPA

## Abstract

Recently, several deep learning methods have been applied to decoding in task-related fMRI, and their advantages have been exploited in a variety of ways. However, this paradigm is sometimes problematic, due to the difficulty of applying deep learning to high-dimensional data and small sample size conditions. The difficulties in gathering a large amount of data to develop predictive machine learning models with multiple layers from fMRI experiments with complicated designs and tasks are well-recognized. Group-level, multi-voxel pattern analysis with small sample sizes results in low statistical power and large accuracy evaluation errors; failure in such instances is ascribed to the individual variability that risks information leakage, a particular issue when dealing with a limited number of subjects. In this study, using a small-size fMRI dataset evaluating bilingual language switch in a property generation task, we evaluated the relative fit of different deep learning models, incorporating moderate split methods to control the amount of information leakage. Our results indicated that using the session shuffle split as the data folding method, along with the multichannel 2D convolutional neural network (M2DCNN) classifier, recorded the best authentic classification accuracy, which outperformed the efficiency of 3D convolutional neural network (3DCNN). In this manuscript, we discuss the tolerability of within-subject or within-session information leakage, of which the impact is generally considered small but complex and essentially unknown; this requires clarification in future studies.

## Introduction

In cognitive neuroscience, the framework for predicting the stimuli given to subjects or the tasks they perform based on their neural activity is called “decoding.” From a modeling perspective, we can evaluate predictive power and identify the brain regions that are the most informative for specific stimuli or tasks. Decoding has also been studied extensively in the context of mind-reading. The most widely used decoding strategy is a pattern classification method called Multi Voxel Pattern Analysis (MVPA; Cohen et al., 2017). Haxby et al. (2001) showed that visual categories of stimuli can be classified based on neural activity, distributed and not clustered in small areas of the ventral temporal lobe. Subsequently, the feasibility of decoding has been explored using a variety of machine learning methods. Typically, these include various types of classifiers such as the logistic regressions, the Support Vector Machine, and the Gaussian Naive Bayes.

More recently, with the increasing interest in deep learning, studies applying non-linear multi-layer network models to decoding have been reported (Koyamada et al., 2015; Gao et al., 2019b; Thomas et al., 2019). Deep learning has the advantage of being able to simultaneously learn end-to-end, overcoming the previous faults of multi-step learning processes; previously, the classifier was learned after extracting brain regions as features, but it has now become possible to perform feature extraction and classifier learning from the whole brain at once (Wang et al., 2020). However, there remain some problems, such as the difficulty in applying deep learning to high-dimensional data and small sample size conditions (Cho et al., 2016; Yang et al., 2017).

In cognitive neuroimaging research, there tends to be a paucity of data due to experimental costs in terms of participant selection or session length, due to the complex demands of such research. When using machine learning for data analysis in sporadic experiments of this type, low statistical power and large errors in the evaluation of predictive accuracy often result. There is no clear solution to this issue, since it is important to exclude any unavoidable information leakage from a within-subject analysis. This is a crucial issue, especially when applied to a clinical context (Varoquaux et al., 2017; Varoquaux, 2018; Cearns et al., 2019).

In this study, in using a small-sized neurocognitive dataset, several cross-validation methods with different split units were used to evaluate the relative fit of different models. The models were used to analyze the results of a neurolinguistic experiment, from which a multi-site large-scale dataset is unlikely to be produced. In detail, we adopted a complicated task design for the experiment (conceptual association involving language switch), with an idiosyncratic subject group (early bilinguals familiar with two heterogeneous orthographic systems). This problem setting is particularly problematic for deep learning models because of the high-dimensional and small sample size dataset. At this point, we also identified the best method to adjust for and minimize information leakage to obtain desirable performance in the presence of a small-sized neurocognitive dataset.

## Methods

This study was performed in accordance with the Declaration of Helsinki and was approved by the Ethics Committee of the Tokyo Institute of Technology (approval number: B13001). Written informed consent was obtained from all subjects before participation. The details of the experiment are described in the Supplementary Material.

### Datasets

Five Korean-Chinese early bilinguals participated in the functional magnetic resonance imaging (fMRI) experiments, which involved six repeated runs of a total of 20 mammal or 20 tool object images with name captions given in either Korean or Chinese, depending on the run numbers. The dataset consisted of 1,200 trials (6 runs × 40 items for each subject; 600 trials for each class) produced by a rapid event-related design with stimulus randomization. For each trial, response data were obtained by using boxcars for 5–8 s after the stimulus onset (Akama et al., 2012); hence, there were four boxcars for which the magnitudes were averaged to generate data in each trial (except for one classifier described below). The target of the group-level MVPA was focused on the discrimination of the conceptual categories (“mammal” versus “tool”), although the language difference could result in a small degree of interference.

Using SPM8 (Friston et al., 1994), we performed a series of pre-processing steps including head movement correction, superimposing anatomical images, gray matter segmentation, conversion to Montreal Neurological Institute (MNI) coordinates, and resolution correction, after which a gray mask was applied using Nipy (Millman and Brett, 2007). Furthermore, each volume was cropped to exclude areas that were not part of the brain before z-scoring the entire image.

### Classifiers

Based on previous studies, we used four classifiers: penalized logistic regression (PLR), support vector machine (SVM), multichannel 2D convolutional neural network (M2DCNN), and 3D convolutional neural network (3DCNN). The codes for PLR and SVM were implemented using the Python package scikit-learn (Pedregosa et al., 2011), while those for M2DCNN and 3DCNN employed Pytorch (Paszke et al., 2019; both available at: https://github.com/sn0422j/mt_deep).

The PLR (L2 norm) and SVM (Linear SVM) were used, respectively, as the most popular classifiers. Regularization parameters were optimized with nested cross-validation (Nested-CV); for the activity vector, the boxcars were averaged, and 500 voxels were selected by analysis of variance (ANOVA).

**FIGURE 1:**
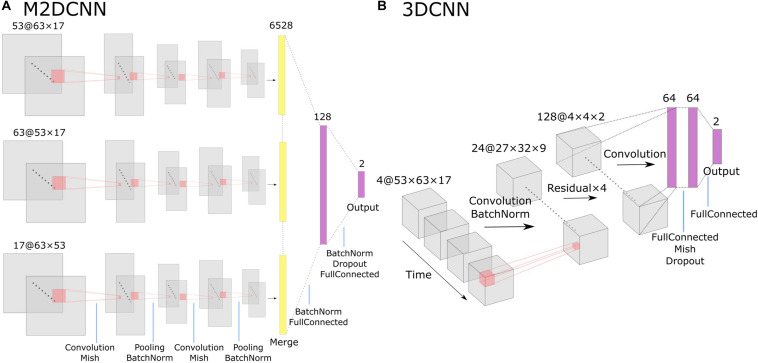
The architecture of Two deep learning models: **(A)** the multichannel 2D convolutional neural network (M2DCNN) model and **(B)** the 3D convolutional neural network (3DCNN) model. Dropout rate of the fully connected layer was set to 0.5 in both **(A)** and **(B)**.

For the M2DCNN model, we referred to the work of Hu et al. (2019), which meant that the model consisted of three two-dimensional convolutional layers corresponding to the axes of three orthogonal planes, a merge layer that concatenates features, and a fully connected layer for classification. Figure 1A shows the architecture of this model. A Mish function (Misra, 2019) was used for the activation function to prevent overfitting. To train our model, we used cross-entropy as a loss function and Adam [learning rate = 0.001, beta = (0.9, 0.999)] for optimization; 300 epochs were performed with exponential learning rate decay. The average images of the boxcars were used as the input to the model.

The 3DCNN model was based on the report of Wang et al. (2020; Figure 1B) to capture local spatiotemporal changes by applying three-dimensional convolutional filters over a time series. This training configuration was the same as the M2DCNN model, and the boxcars were used as the input to the model. It should be noted that the 3DCNN model allowed us to input without averaging the magnitudes of the critical boxcars as a single 4D data.

### Evaluation of Accuracy

With a view to comparing the effectiveness of the following three cross-validation (CV) methods, we performed a five-fold CV for each method to calculate the classification accuracy of the test set split out from the small boxcar data: leave-one-subject-out CV, session shuffle split, and sample shuffle split. Note that for these CV methods, the data for the folds were subtracted from the six runs in each experiment, since we did not leave out any run(s) as a unit in this modeling.

**FIGURE 2:**
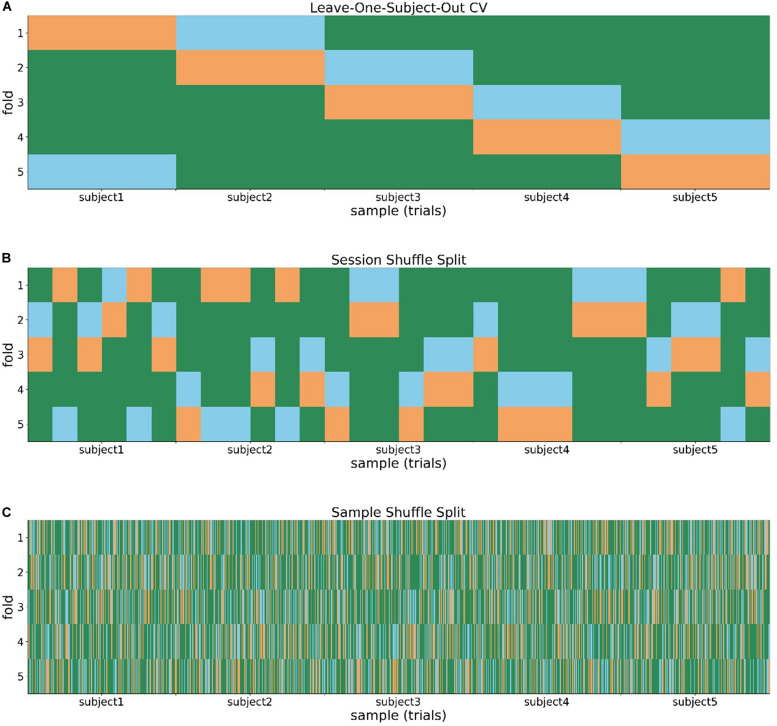
Three splitting methods for the evaluation of accuracy: **(A)** Leave-one-subject-out cross-validation; **(B)** session shuffle split; and **(C)** sample shuffle split. The training set (green), validation set (blue), and test set (orange) are colored differently.

When using the leave-one-subject-out CV as a splitting strategy, each subject was assigned a particular fold pattern so that only one individual’s data was included in each test set (abbreviated hereafter as Test) and another one in the validation set (abbreviated as Valid) at every CV step (Figure 2A). Hence, each fold contained three subjects as providers of a training set (Train for short), one subject for the Valid, and another one for the Test.

In the session shuffle split, a fold was created in a run-by-run manner, regardless of subject identification, and by selecting 20% of the trials (taken as blocks) included in each run as Test or Valid at random (Figure 2B). The proportion of the numbers of data randomly assigned to the Train, Valid, and Test sets was identical throughout all folds (3:1:1). In other words, in each fold we had 18 runs for Train, 6 runs for Valid, and 6 runs for Test.

In the sample shuffle split method, a fold was set in a trial-by-trial manner, without considering data attribution to subject and runs, and by randomly selecting 144 trials for Train, 48 trials for Valid, and the remaining 48 trials for Test (Figure 2C). The proportion of the three subsets was kept at 3:1:1 as was the case with the session shuffle split method.

Additionally, we performed a permutation test in which labels were randomly re-labeled 100 times, to calculate the chance level for each cross-validation method. With regards to the classifier, the PLR with the above settings was used as the baseline. We calculated the *p* value for each combination of classifiers and CV methods using the Wilcoxon rank-sum test. The statistical analyses were conducted using Scipy.stats Version 1.4.1. A *p* value of less than 0.005 was the threshold for statistical significance.

## Results

**FIGURE 3:**
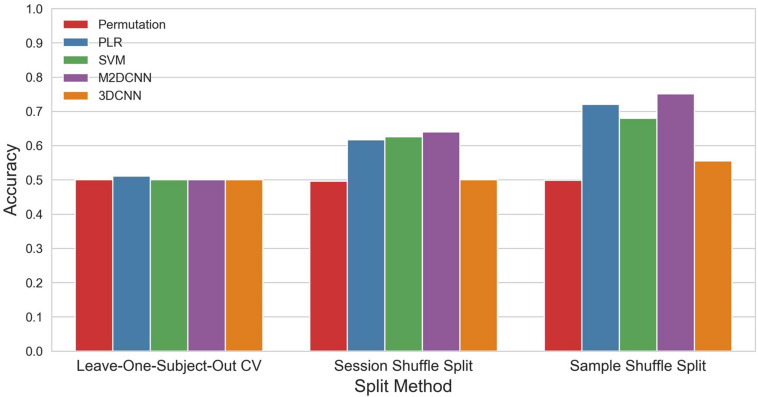
The mean accuracy for each split method and classifier. The permutation accuracy represents the chance level for each split method. 3DCNN, three-dimensional convolutional neural network; M2DCNN, multichannel two-dimensional convolutional neural network; PLR, penalized logistic regression; SVM, support vector machine.

The accuracy and *p* values for the three cross-validations and the four classifiers are shown in Figure 3 and Table 1. PLR, SVM, and M2DCNN elicited significantly higher accuracy (*p* value < 0.0005) than the chance level for the session shuffle split and sample shuffle split. 3DCNN recorded almost the same accuracy as the chance level.

**TABLE 1:** The mean accuracy and the associated *p* value for each split method and classifier.

Split method	Training method	Accuracy	*p* value
Leave-One-Subject-Out CV	PLR	**0.511**	0.1529
	SVM	0.500	0.9760
	M2DCNN	0.500	0.9700
	3DCNN	0.500	0.9700
Session Shuffle Split	PLR	0.617	0.0002**
	SVM	0.626	0.0002**
	M2DCNN	**0.640**	0.0002**
	3DCNN	0.500	0.9101
Sample Shuffle Split	PLR	0.720	0.0002**
	SVM	0.680	0.0002**
	M2DCNN	**0.751**	0.0002**
	3DCNN	0.555	0.0017*

A single asterisk (*) indicates *p* < 0.005; double asterisks (**) indicate *p* < 0.0005. CV, cross-validation. The highest accuracy in each split method is highlighted in bold.

In general, the accuracy of the classifier was improved with, in ascending order, leave-one-subject-out CV, session shuffle split, and sample shuffle split. In the leave-one-subject-out CV, the best precision rate (0.511) was obtained with the PLR classifier, but this was not significant (*p* value > 0.005). In the session shuffle split and sample shuffle split, the best precision rate (0.640, 0.751, respectively) was obtained with the M2DCNN classifier. We regarded the value of 0.640 using session shuffle split as the authentic accuracy, which is discussed below.

## Discussion

### Comparison of Methods

In this section, we review the properties of all of the split methods, respectively, and then assess the performance and structure of several deep learning models. Each CV method has a different data distribution within each of the splits. For the leave-one-subject-out CV, the breakdown of a fold composed of Train, Valid, and Test was limited to 3, 1, and 1 subject(s), respectively. Thus, we assume that the classifiers would be insufficient to achieve good generalization performance in the classification of further unknown subjects. The data were under-sampled from a large population of subjects, since the individual variability between subjects should be significantly larger than within-subject fluctuations in terms of functional activity (Miller et al., 2009). As a result, statistical machine learning methods were far from a good fit.

When it comes to the session shuffle split and sample shuffle split methods, for which within-subject leakage was unpreventable, statistical machine learning was likely to be successful by reducing the effect of individual functional differences. Further improvement in the accuracy of the sample shuffle split may be dependent on the leakage caused in a time series due to the higher similarity of functional activity within runs than between runs (Varoquaux et al., 2017; Varoquaux, 2018). Moreover, it should be considered that two types of session-wise stimuli were provided to each subject with orthographic variability by language switch, which might have had a significant impact on his/her task performance.

Individual functional differences have traditionally limited the application of classifiers; solutions addressing this include functional alignment (hyperalignment; Haxby et al., 2011), the use of large datasets (Varoquaux et al., 2017; Varoquaux, 2018), and some few-shot learning techniques like transfer learning in deep learning (Gao et al., 2019a,b; Wang et al., 2020), transfer learning in shared response modeling (Zhang et al., 2018; Yousefnezhad et al., 2020), and meta-learning. It is difficult, however, to use these methods for deep learning with a limited sample size and a unique experimental condition. Therefore, leaking information to some extent as referenceable prior knowledge and discussing end-to-end models appears to be one of the better solutions to address this issue. The session shuffle split model appears to be the best way to evaluate the accuracy of the models in this case, since the individual functional differences are referenceable without being affected by the time-series correlation.

The M2DCNN model, which achieved the highest accuracy with little information leakage, was evaluated using gradients to locate what the model learned for classification. This analysis resulted in consistency with prior research describing similar experimental tasks (see Supplementary Material for analytical details). In this regard, a deep learning end-to-end model could detect category-specific responses that are common to the subjects.

The unexpectedly poor efficiency of the 3DCNN model for the present analysis is worthy of discussion. Prior studies that applied the 3DCNN model to task fMRI (Hu et al., 2019; Wang et al., 2020) showed high accuracy in block designs, with sustained and homogeneous task characteristics. The rapid event-related design that we employed in our experiment might promote greater variability within the time series. Given this, a model that explicitly incorporates time series information, such as long short-term memory (LSTM), may fit better (Thomas et al., 2019) for a checkered experiment session.

### Limitations and Future Directions

In this section, we provide some limitations of this study and discuss the best method to adjust the information leakage level. There are some limitations to this study. The accuracy reported in this study is not an indicator of the generalizable performance of the entire subject population, due to the leakage of information. Here, we define information leakage as the phenomenon where the i.i.d. split units for each split strategy have dependence as a consequence of the structured property of data distribution. There are several levels of information leakage, which should be separated out in terms of legitimacy (Kaufman et al., 2012). Based on this idea, we propose readily attributing levels as “heavy” or “light” for those actions.

In the fMRI decoding framework, heavy leakage has been considered to be so serious that it affects the authenticity of accuracy indicators, such as that seen in supervised feature selection prior to splitting or hyperparameter optimization with Test data (Kaufman et al., 2012). In contrast, light leakage is likely to occur when the Train and Test data are not completely independent, with their indirect and hidden relationship being difficult to scrutinize; its impact is generally taken as small but complex and essentially unknown.

In this study, we presented an example of training a complex model by allowing light information leakage. For group analysis, the sample shuffle split method ignored the leakage likely to be caused in a time series and hypothesized the independence of trials within runs. We believe that under this condition, the rate of 0.651 obtained by the session shuffle split and the M2DCNN classifier was the authentic limit of classification accuracy in this study. Beyond this scope, some results of multivariate analysis based on heavy leakage might be considered to work entirely outside the context of machine learning; for example, an adaptive reuse of them is possible, such as that seen with a brain semantic map reflecting the representational similarity of concepts. However, open questions remain unanswered in relation to the utility of such rich information handling.

In regards to the underestimation of cross-validation loss, our research indicated a need to demonstrate how we could control the data independence and support the significance of the indicator in a non-parametric way; for example, by using a permutation test (Varoquaux et al., 2017; Varoquaux, 2018). However, when investigating cognitive processes specific to a narrow population as in the case of this study, it is important to model within-subject variability by taking more data, even with fewer subjects, and reducing within-subject errors (Smith and Little, 2018). Future studies are required to develop and train a more reliable classifier for each subject and to stably as well as precisely detect consistent shared effects across subjects with higher statistical power.

## Conclusion

In this study, we examined the application of complex models for the decoding of fMRI under the constrained condition of a small sample size in a unique cognitive experiment. It was shown that even when data bias was caused by functional variability across subjects, in spite of greatly limited performance of the classifiers, the complex model could be successfully applied by taking a moderate split to control information leakage. This might be a key to success in deep learning for overcoming a paucity of fMRI data. In this study, we have discussed the tolerability of within-subject or within-session information leakage, of which the impact was generally considered to be small but complex and essentially unknown; this requires clarification in future studies.

## Data Availability Statement

The raw data supporting the conclusions of this article will be made available by the authors, without undue reservation.

## Ethics Statement

The studies involving human participants were reviewed and approved by the Ethics Committee of the Tokyo Institute of Technology (approval number: B13001). The patients/participants provided their written informed consent to participate in this study.

## Author Contributions

SY and HA: conceptualization, investigation, methodology, and writing. SY, ML, and HA: data curation and analysis. HA: supervision. All authors contributed to the article and approved the submitted version.

## Conflict of Interest

The authors declare that the research was conducted in the absence of any commercial or financial relationships that could be construed as a potential conflict of interest.

## Abbreviations

3DCNN: three-dimensional convolutional neural network
ANOVA: analysis of variance
CV: cross-validation
FWHM: full width at half maximum
Leave One Subject Out: leave-one-subject-out cross-validation
M2DCNN: multichannel two-dimensional convolutional neural network
MNI: Montreal Neurological Institute
MVPA: multi voxel pattern analysis
Permutation: permutation test
PLR: penalized logistic regression
SVM: support vector machine
Test: test set
Train: training set
Valid: validation set.
