# Supplementary material for: Evaluation of Task fMRI Decoding With Deep Learning on a Small Sample Dataset
Source: Front Neuroinform. 2021 Feb 12;15:577451. doi: 10.3389/fninf.2021.577451 (PMC7928289; doi:10.3389/fninf.2021.577451)
Supplement: Supplementary file 1 [file Data_Sheet_1.docx]

# Supplementary Materials

**1 Supporting Information for the Materials and Methods**

**1.1 Task Design**

The design of the fMRI experiment that provided the data for this study was identical to that of the studies published by Akama et al. (2012) and Lei et al. (2015). It adheres fundamentally to the orthographic condition of Akama et al. (2012), except that two independent factors were interlaced with each other across the six total runs: conceptual categories and kinds of language. The research result originally assuming a double-target MVPA has not been published previously, and none of the data were dealt with in our subsequent articles.

In the present study, a slow event-related design was used. Five Korean-Chinese bilinguals fluent in both languages participated in the fMRI assessments. They were shown a total of 40 contrast-normalized grey-scale photographs of 20 mammal or 20 tool objects with captions indicating the object names in either Korean or Chinese, depending on the runs. In one trial, each concept was presented for 3,000 ms, followed by a fixation cross for 7,000 ms in the center of the screen. The participants were requested, during a 3,000 ms stimulus time, to perform a covert property generation task using the same language as that of the caption, by thinking of the appropriate features of the given concept. In each run, one half of the objects, representing 10 mammals and 10 tools, were randomly provided using Korean language, while the other half of the remaining objects were provided in Chinese. Throughout the experiment, the language describing a concept was switched, with every run keeping the same concept subgroups, including the two concept categories. Hence, the captions as orthographic information were identical across the even or the odd runs, but not across both. For example, the concept “giraffe” in subgroup 1 was presented with a Chinese caption in the first, third and fifth runs, and with Korean captions in the second, fourth and sixth runs, whereas the inverse was true for the concept “power drill” in subgroup 2. Due to this assignment of independent factors, language switches taking place at unexpected times gave rise to noise for the property generation task; the stimulus sets bound to the runs were not homogenous and thereby made cross-validation of classificatory model biased and more challenging. The list of 40 concepts is given below (written in Chinese, English and Korean):

Mammals: anteater (食蚁兽，개미핥기), armadillo (穿山甲， 아르마딜로), beaver (河狸, 비버), camel (骆驼，낙타), deer (鹿，사슴), elephant (大象，코끼리), fox (狐狸，여우), giraffe (长颈鹿，기린), gorilla (大猩猩，고릴라), hare (野兔，토끼), hedgehog (刺猬，고슴도치), hippopotamus (河马，하마), kangaroo (袋鼠，캥거루), koala (考拉熊，코알라), mole (地鼠， 두더지), monkey (猴子， 원숭이), panda (熊猫， 참대곰), rhinoceros (犀牛，코뿔소), skunk (臭鼬鼠，스컹크), zebra (斑马，얼룩말).

Tools: Allen key (六角匙，앨런 볼트용 렌치), axe (斧头，도끼), chainsaw (链锯，동력 사슬톱), craft knife (工艺刀，다용도 칼), file (锉刀，줄), hammer (铁锤，망치), nail (钉子，못), paint roller (油漆滚筒，페인트 롤러), trowel (抹泥刀，모종삽), pliers (钳子，펜치), plunger (活塞， 플런저), power drill (电钻， 동력 천공기), rake (耙子，갈퀴), saw (锯子，톱), scraper (刮刀， 긁어내는 도구), scissors (剪刀，가위), screw (螺丝钉，나사), sickle (镰刀，낫)，spanner (扳手，스패너), tape measure (卷尺，줄자).

**1.2 Data Acquisition**

Functional MRI scans were performed using a 3.0 T SIGNA MRI scanner (GE Healthcare, Chicago, IL, USA) at Tokyo Institute of Technology, Japan with an 8 channel high resolution head coil. Scanning parameters were based on those of Mitchell et al. (2008). Functional scanning was performed using an echo planar imaging sequence with a 1,000 ms repetition time (TR), 30 ms echo time (TE), and 60 degree flip angle (FA). Each volume consisted of 15 × 6 mm thick slices with an interslice gap of 1 mm; field of view (FOV): 20 × 20 cm; size of acquisition matrix, 64 × 64; number of excitations (NEX): 1.00. The parameter values of the anatomical scans were TR=7.284 ms, TE=2.892 ms, FA=11 degrees, band width=31.25 kHz, and voxel size=1 mm isotropic. Following settings used by Mitchell et al. (personal communication), we set oblique slices in the sagittal view with a tilt of -20 to -30 degrees such that the most inferior slice was above the eyes anteriorly and passed through the cerebellum posteriorly.

**1.3 Accuracy of Within-Subject Classification**

A within-subject binary classification was performed. We used the support vector machine (Linear SVM) as the classifier, and leave-one-run-out cross-validation was used to evaluate the accuracy. The mean accuracy was 0.78 for one subject. This performance is comparable to that of previous studies with a similar experimental design (Akama et al., 2012; Kampa et al., 2014; Chen et al., 2020). Thus, we believe that this dataset was of good quality for within-subject classification.

**2 Supporting Information for the Discussion**

**2.1 Understanding Deep Learning Models**

The M2DCNN model, which was learned end-to-end from the whole brain without feature selection beforehand, was evaluated with integrated gradients (Sundararajan et al., 2017). This method uses gradients to understand the relative importance of input features in a deep learning network. In this article, we analyze the results of the session shuffle split, which was the most successful in learning with little information leakage. Integrated gradients were obtained from the test data of the best-accurate combination of fold and subjects, and Cohen's d (Cohen, 1988) was computed for each voxel between the two categories of “mammal” and “tool”. After smoothing with a full width at half maximum (FWHM) of 6 mm, voxels with an absolute value of d > 0.8 were identified (Table 2 and Figure 4).


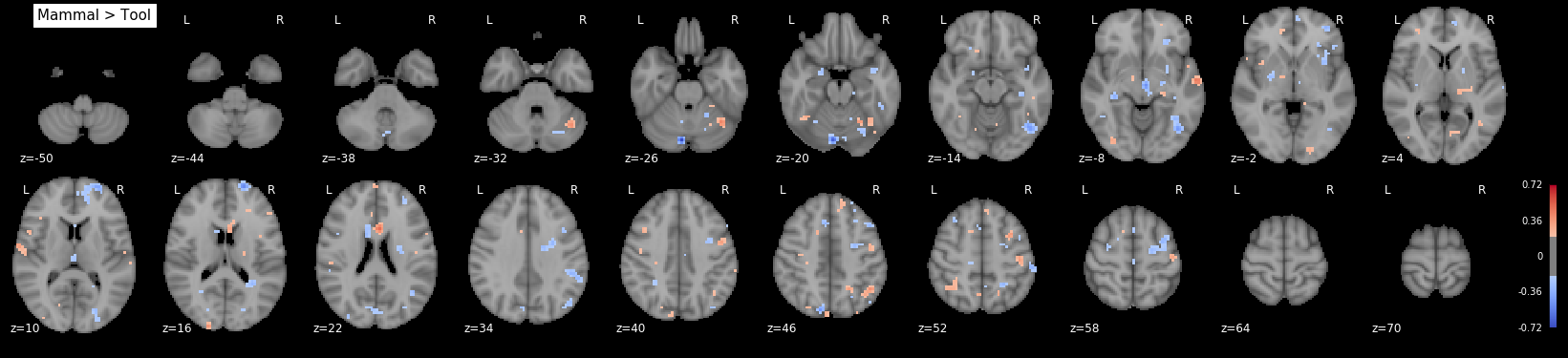


Figure 4. Visualization of a brain map via Cohen’s d effect sizes (mammal > tool) with integrated gradients (full width at half maximum [FWHM] = 8 mm). The positive effects are colored in red, and the negative ones are colored in blue. The threshold was manually selected for visualization effects, and the brain map was normalized to Montreal Neurological Institute (MNI) coordinates.

Table 2. Highly effective regions according to the integrated gradients


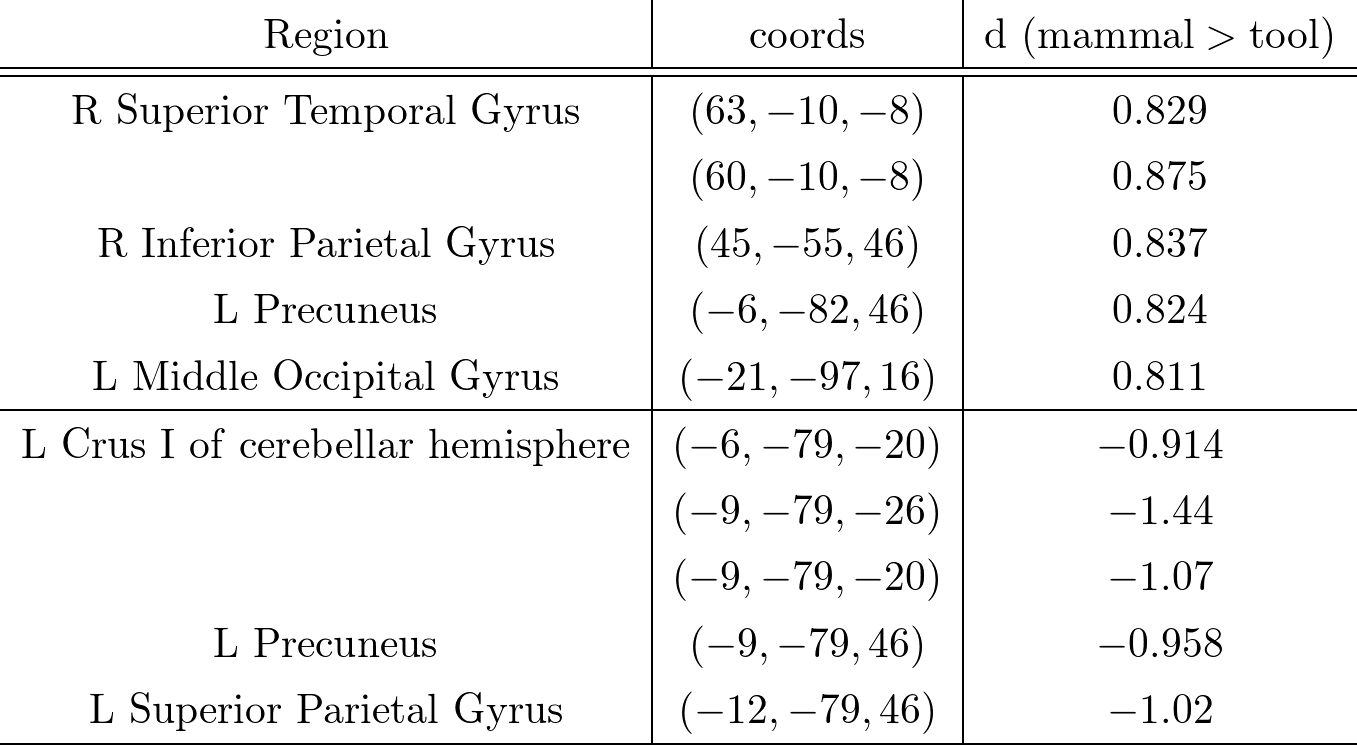


The coordinates are in MNI space, and the name of the region is derived from the Automated Anatomical Labelling (AAL) atlas (Rolls et al., 2020). Coords: coordinates; L: left; R: right.

This result was consistent with previous research in that "mammal" words were more responsive to the right temporal and the left occipital cortices, whereas "tool" words tended to recruit the left temporal cortex (Pulvermüller, 2001; Akama et al., 2012). On the other hand, the left precuneus, which is recognized as a central node of the default-mode network (DMN), showed important effect sizes for the processing of both semantic categories. It is noteworthy that this region is known to have the ability to let subjects internally focus on cognitive process and integrate the other functional networks, which may reflect subject-specific activity patterns during the task performance (Utevsky et al., 2014.). In light of this, our model appears to be able to discriminate the category-specific responses shared across the subjects, and subject-specific information also contributes to improve the precision of the model.

**2.2 Results and Replicability for the Dataset of Akama et al. (2012)**

The dataset of Akama et al., 2012 was different from that of the current study only in the used language (Japanese), task complexity (without any language switching) and the participants’ speaking languages (Japanese monolinguals). It would be also useful to reinforce the effectiveness of our new methods. We found that this dataset was useful for replication studies as noted below.

In the dataset of Akama et al. (2012), the accuracy and p values for the three cross-validations and the four classifiers are shown in Figure 5 and Table 3. These figures and tables correspond to Figure 3 and Table 1 in the main part. It turns out that the M2DCNN with the session shuffle split and the sample shuffle split also recorded accuracies which were comparably significant for the dataset of Akama et al. (2012). This result is consistent with the main finding that M2DCNN has the same performance as less complex models under authentic accuracy with little information leakage. However, the task design of Akama et al. (2012), which is simpler than the current one, might homogeneously maintain the substance of the sessions and samples as targets of shuffling. Therefore, the dataset of the current study would be more efficacious than that of Akama et al. (2012) to underscore the important point, which is the session/sample shuffle split.


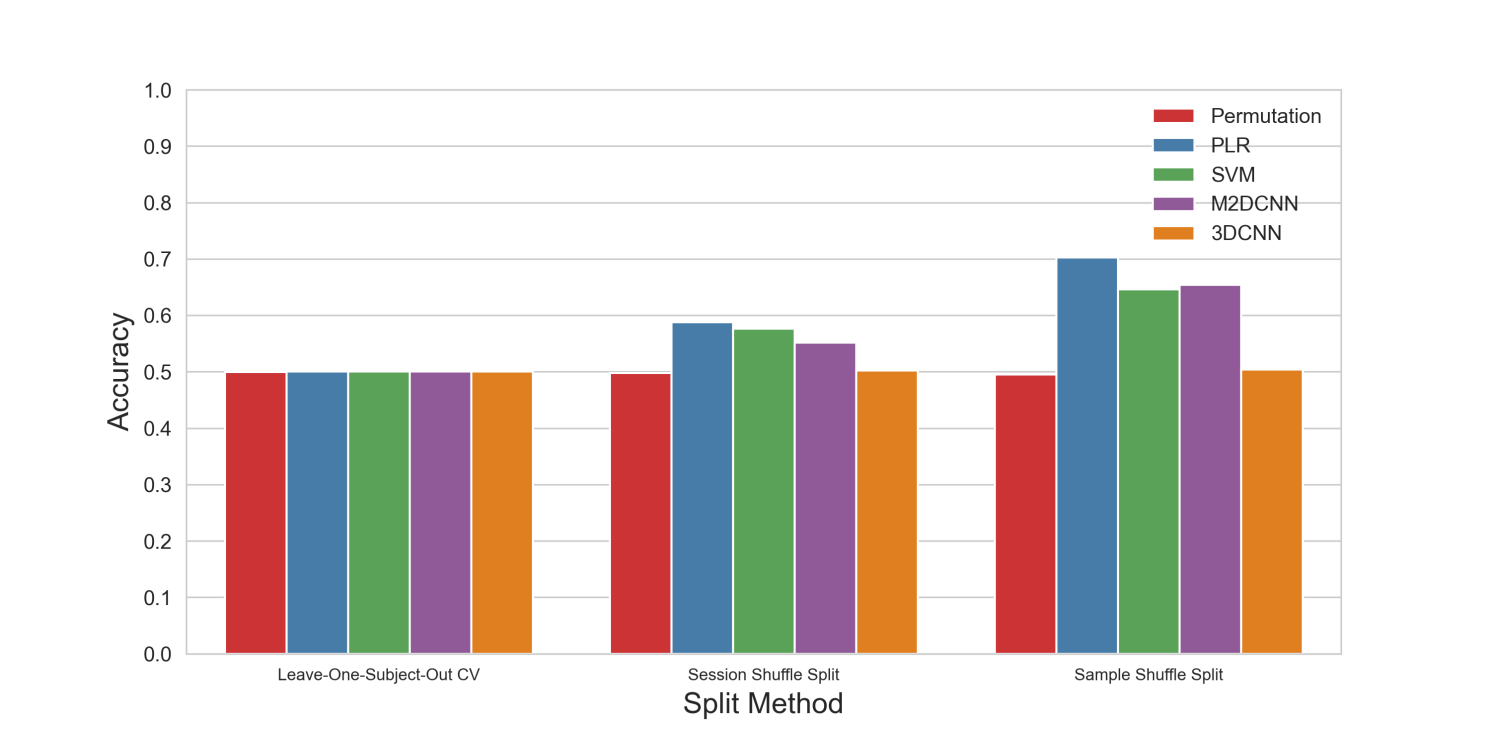


Figure 5. The mean accuracy for each split method and classifier in the dataset of Akama et al. (2012). The permutation accuracy represents the chance level for each split method. 3DCNN: three-dimensional convolutional neural network; M2DCNN: multichannel two-dimensional convolutional neural network; PLR: penalized logistic regression; SVM: support vector machine.

Table 3. The mean accuracy and the associated p value for each split method and classifier in the dataset of Akama et al. (2012)


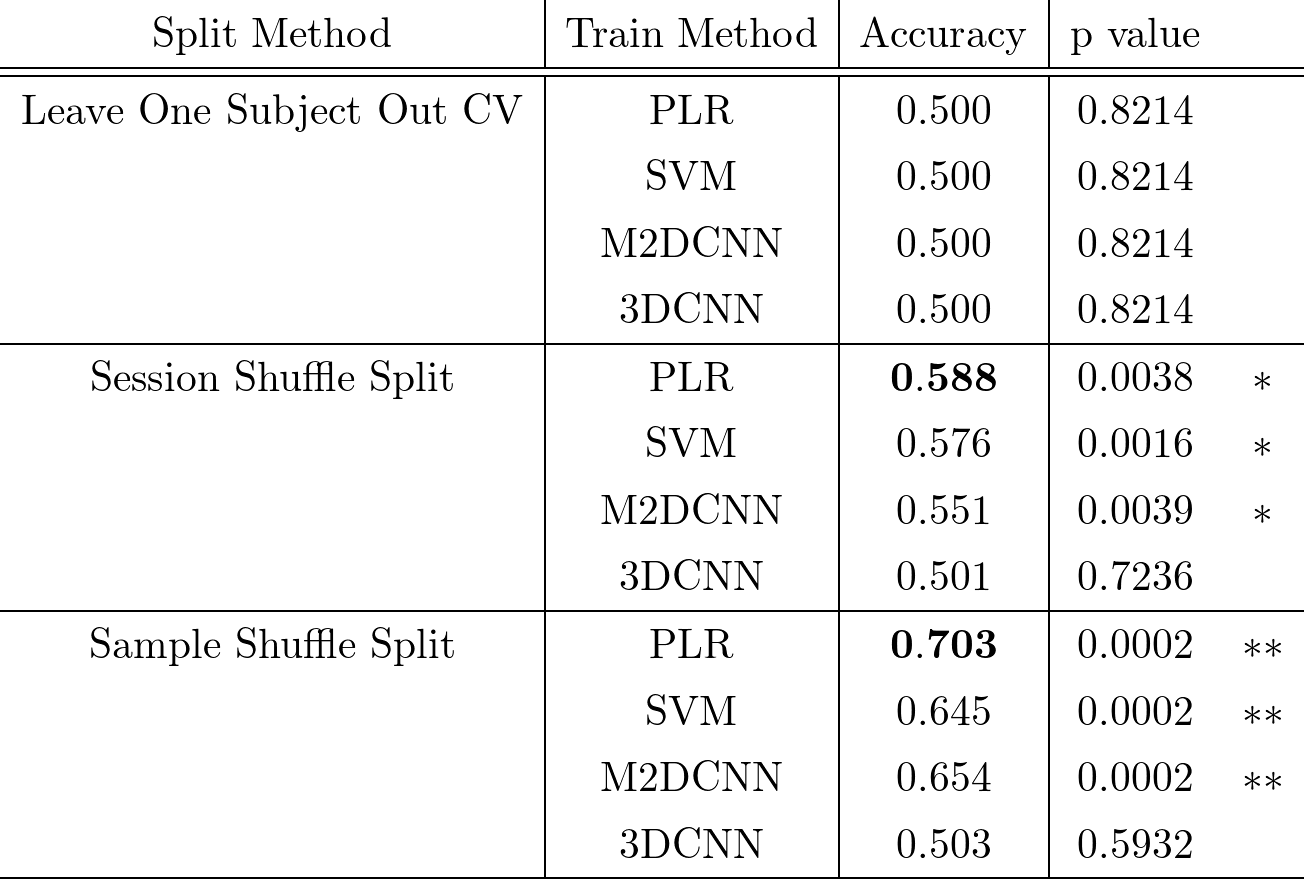


A single asterisk (*) indicates p <0.005; double asterisks (**) indicate p <0.0005. CV: cross-validation.

**3 References**

Chen, C., Batselier, K., Yu, W., Wong, N. (2020). Kernelized support tensor train machines. arXiv:2001.00360. <https://arxiv.org/abs/2001.00360>

Cohen, J. (1988). Statistical power analysis for the behavioral sciences, 2nd ed. Hillsdale, NJ: L. Erlbaum Associates.

Kampa, K., Mehta, S., Chou, C.A., Chaovalitwongse, W.A., Grabowski, T.J. (2014). Sparse optimization in feature selection: application in neuroimaging. J. Glob. Optim. 59, 439–457. <https://doi.org/10.1007/s10898-013-0134-2>

Lei, M., Akama, H., Murphy, B. (2014). Neural basis of language switching in the brain: fMRI evidence from Korean–Chinese early bilinguals. Brain Lang. 138, 12–18. <https://doi.org/10.1016/j.bandl.2014.08.009>

Mitchell, T.M., Shinkareva, S.V., Carlson, A., Chang, K.M., Malave, V.L., Mason, R.A., et al. (2008). Predicting human brain activity associated with the meanings of nouns. Science 320, 1191–1195. <https://doi.org/10.1126/science.1152876>

Pulvermüller, F. (2001). Brain reflections of words and their meaning. Trends Cogn. Sci. 5, 517–524. <https://doi.org/10.1016/S1364-6613(00)01803-9>

Rolls, E.T., Huang, C.C., Lin, C.P., Feng, J., Joliot, M. (2020). Automated anatomical labelling atlas 3. Neuroimage 206, 116189. <https://doi.org/10.1016/j.neuroimage.2019.116189>

Sundararajan, M., Taly, A., Yan, Q. (2017). Axiomatic attribution for deep networks. arXiv:1703.01365. <https://arxiv.org/abs/1703.01365>

Utevsky, A.V., Smith, D.V., Huettel, S.A. (2014). Precuneus is a functional core of the default-mode network. J. Neurosci. 34, 932–940. <https://doi.org/10.1523/JNEUROSCI.4227-13.2014>
